# FAMILY HEALTH STRESS AND INSOMNIA SYMPTOMS AMONG OLDER ADULTS: THE ROLE OF RELATIONSHIPS WITH THEIR CHILDREN

**DOI:** 10.1093/geroni/igae098.4188

**Published:** 2024-12-31

**Authors:** Jeein Jang

**Affiliations:** University of Massachusetts Boston, Quincy, Massachusetts, United States

## Abstract

Insomnia symptoms that arise from stress pose a significant physical and psychological health concern for older adults. Yet, little empirical research has explicitly examined the association between stress caused by family health concerns and insomnia symptoms, along with the role that relationships with children play in moderating this relationship. This study uses Pearlin’s stress process model and data from the 2018 and 2020 Health and Retirement Study (N=7,755) to investigate these associations in adults aged 50 and above. Ordered logistic regressions assess the link between family health stress and insomnia severity using four items (i.e., trouble falling asleep, nighttime awakening, early morning awakenings, and feelings of nonrestorative sleep). Additionally, the moderating role of two relationships with children, positive and negative, is included in the analysis. All statistical analyses are adjusted for sociodemographic, health-related factors, caregiver status, and their children’s socioeconomic status. Results show older adults experiencing ‘somewhat upsetting’ and ‘very upsetting’ family health stress had 24% and 50% higher odds, respectively, of experiencing more severe insomnia symptoms compared to those with no family health stress. Interestingly, both positive and negative relationships only moderated the association between ‘somewhat upsetting’ stress and insomnia symptoms. These findings underscore the need for targeted clinical and public health interventions to help older adults with family health stress reduce their insomnia symptoms by fostering positive relationships with their children.

